# Raising Children in Risk Neighborhoods from Chile: Examining the Relationship between Parenting Stress and Parental Adjustment

**DOI:** 10.3390/ijerph19010045

**Published:** 2021-12-21

**Authors:** Eduardo Sandoval-Obando, Marta Alcaide, Miguel Salazar-Muñoz, Sebastián Peña-Troncoso, Claudio Hernández-Mosqueira, Sofia Gimenez-Serrano

**Affiliations:** 1 Escuela de Psicología, Instituto Iberoamericano de Desarrollo Sostenible, Facultad de Ciencias Sociales y Humanidades, Universidad Autónoma de Chile, Temuco 4810101, Chile; eduardo.sandoval@uautonoma.cl; 2 Department of Methodology of Behavioral Sciences, Faculty of Psychology, University of Valencia, 46010 Valencia, Spain; maralna2@alumni.uv.es; 3 Faculty of Psychology, Universidad San Sebastián, Puerto Montt 5480000, Chile; miguel.salazar@uss.cl; 4 Instituto de Ciencias de la Educación, Universidad Austral de Chile, Valdivia 5110566, Chile; sebastian.pena@uach.cl; 5 Facultad de Educación y Cultura, Universidad SEK, Santiago 5110566, Chile; 6 Departamento de Educación Física, Deportes y Recreación, Universidad de La Frontera, Temuco 4780000, Chile; 7 Department of Developmental and Educational Psychology, Faculty of Psychology, University of Valencia, 46010 Valencia, Spain; sogise@alumni.uv.es

**Keywords:** parenting, parental stress, depression, anxiety, childhood, development

## Abstract

Introduction: Parenting stress and parental adjustment could implicate key differences in the relational dynamics that parents establish with their children, particularly when families come from vulnerable social contexts. Method: Participants were 142 fathers and mothers from a risk neighborhood of Chile. The variables examined were parenting stress (parental distress, parent–child dysfunctional interaction and difficult child) and parental adjustment (depression, anxiety, and stress). Parents also completed a sociodemographic characterization survey. The statistical analyses were a correlation analysis and multiple linear regression analyses. Results: Overall, not all components of parenting stress were related to parental adjustment. Only parental distress was found as a significant predictor of poor parental adjustment (greater depression, anxiety, and stress), but not parent–child dysfunctional interaction and having a difficult child. Conclusions: The present study findings highlight the influence of stress on parenting as a relevant dimension of research for the improvement of the intervention deployed by the state regarding the protection of vulnerable Chilean children, providing multiple clinical and psychosocial applications for research and intervention purposes.

## 1. Introduction

Risk neighborhoods are those communities with greater poverty, delinquency, unemployment, violence, or drug use, in comparison to middle-class neighborhoods [1,2,3]. Parents’ main responsibility is raising children [4,5]. However, opportunities for child and adolescent healthy development could be lower in risk neighborhoods than in middle-class neighborhoods [6,7]. In risk neighborhoods, there is a greater risk of problematic development; children and adolescents could be less mature, less competent, and more prone to internalized symptoms and to externalizing problems [3,6,8].

There is a consensus that the work of being a father is a complex task that involves the development of competencies at different levels for an adequate achievement of the parental function [9,10,11]. In recent years, support programs for families in contexts of psychosocial risk have become an important focus in the design of child protection systems, largely justified by: (a) the high level of child abuse [12,13,14]; (b) the evidence supporting early family intervention in contexts of psychosocial risk [15,16,17]; (c) the multiple studies on adverse experiences in childhood and their impact on human development [18,19,20] and (d) the gradual social recognition of children’s rights [13,21].

Overall, the types of support that programs provide to parents are varied, among which can be found, for example, those that focused on providing information and guidance on parental exercise or the delivery of social supports to overcome difficulties, reducing parental stress, and helping families that are usually associated with inadequate educational access [12,22]. Thus, the importance of preventive programs lies in reducing or avoiding the need for more expensive and less effective secondary programs, in addition to preventing violence and child abuse [15,21].

Thus, targeted prevention programs (PPF), as part of the public policy focused on vulnerable children in Chile, seek to intervene in situations of moderate violations of rights. These situations are related to the family context, affecting children and adolescents (NNA) under eighteen years of age, who do not necessarily require separation from their family (mild-to-moderate psychological abuse; witness of domestic violence not constituting a crime; mild-to-moderate physical abuse without current complaints to the prosecutor’s office or police; moderate non-chronic neglect, among others). Therefore, targeted prevention programs (PPF) are, in practice, a relevant device to mitigate some of the effects of early deprivation at the level of violation of rights towards children and adolescents and, potentially, to improve the quality of life of the families admitted [23,24,25].

At this point, the mental health of caregivers was considered an important variable that could affect the balance of family functioning [9,26,27]. In addition, various studies linked high levels of stress associated with parenting to deterioration in caregiving skills, high rates of child abuse, and negative results in child development and family dynamics [28,29,30]. Despite this, some investigations, which analyzed the functioning of PPF in the Chilean context reflected the absence of the mental health dimension as a focus of promotional and preventive work, an aspect that contradicts with the international evidence in this field [23,24,25,31].

The present work aims to examine the level of mental health and psychosocial stress in caregivers who receive support in the Focused Prevention Programs of the National Service for Minors in the Los Lagos Region (Chile).

### 1.1. Targeted Prevention Program: Areas of Intervention with Families in Vulnerable Contexts

In Chile, the National Service for Minors is the body in charge of executing the special public policy of supporting children and adolescents whose rights are violated in adoption processes and in conflict with the law. Within the outpatient programs in the field of the protection of children’s rights are the PPF, which, for the year 2019, covered 41.7% (56, 131) of the total care of outpatient programs in the protection of rights, ranking it as the program with the highest level of care in Chile [32]. The intervention is organized from an ecological perspective, in which the children and adolescents are seen to be part of a family and socio-community environment, and thus is carried out in an articulated way to overcome the situation that warranted entry into the program. This is carried out through actions that aim to strengthen the personal resources of children and adolescents according to their stage of development. These actions include strengthening the parental competencies and resources of the adults in charge that favor the restitution of the violated rights, as well as promoting the incorporation of co-guarantors from the family and community environment that contribute to the restitution of the rights of children and adolescents by supporting parents (primary caregivers) in the upbringing of their children [32].

In general, there is an international trend of developing family support programs to achieve the exercise of parenting that provides quality care for children and adolescents [12,22]. This was supported by meta-analyses that reported positive effects on children’s emotional and behavioral difficulties in selective prevention and specialized treatment programs [33] for reducing child maltreatment [12] and developing parenting attitudes and positive parenting practices [21]. Despite this, in the South American context, evidence-based programs for parents at psychosocial risk tend to be scarce, generating a significant gap between the available research and the intervention practices that are developed in many parenting support programs.

### 1.2. Parental Mental Health and Quality of Care

There is enough reported evidence that the presence of depressive symptoms in mothers can affect the capacity for a dyadic and triadic alliance with their children [26,34], and high levels of stress causes less availability to respond to children’s needs [12,22]. Although fathers have been studied less, there is also research that reports that the presence of high levels of stress [35] and depressive symptoms in parents [36] negatively affects the quality of parent–child interactions.

Parental stress is a specific type of stress that occurs in response to the specific de-mands of parenting, which in turn can be related to other types of stress within the family context, such as stress caused by social or economic factors [37]. It is an activating element that promotes the use of available resources to satisfactorily face the parental role, the absolute lack of activation being as damaging as extreme levels of stress [38].

The available research described that a large part of the families that are in a sit-uation of psychosocial risk tend to present high levels of tension associated with the exercising of their role as parents [15,17,39,40]. Recently, Santelices et al. (2021) [41] found, in a Chilean sample of 123 mother–child dyads with a low socio-economic level, a positive relationship between stress in mothers with greater difficulties and the socioemotional development of their children of preschool age. Although a direct association between depressive symptoms and social-emotional development in children was not found, it was observed that the symptoms of depression and parental stress were closely related, especially in the management of children’s behavior due to their own characteristics.

The relationship between stress and depression in fathers and mothers was de-scribed in different investigations [42,43], as well as the impact of stress on the socio-emotional development of children [44,45]. In Chile, a recent analysis carried out by UNICEF, [40] based on data from the Longitudinal Survey of Early Childhood, a direct association between psychological factors of caregivers with child development in the socio-emotional and cognitive sphere was reported. Specifically, parental stress was the factor most frequently associated with the development of children in various areas, while mental health (specifically depressive symptoms) also appeared as a factor that affected socio-emotional development.

### 1.3. Present Study

Overall, in previous studies the negative consequences for health and wellbeing associated with parental stress were identified [46,47,48,49]. Nevertheless, most studies examined the harmful consequences of parenting stress based on a unidimensional (i.e., global) approach [46,47,48]. Parenting stress, as a global concept, is only assessed if related to adjustment criteria; however, it is not possible to identify which component of parenting stress (e.g., parental distress, parent–child dysfunctional interaction and difficult child) is related to each adjustment criteria. By contrast, the use of a multidimensional approach is less common in studies on parenting stress [49,50]. Interestingly, some of these previous studies suggested that not all components of parenting stress could be related to adjustment criteria, although evidence is mostly focused on legal criteria such as parental physical abuse and other forms of maltreatment [50], or child adjustment criteria such as child oppositionality and internalizing and externalizing problems in children [49].

Additionally, the literature regarding parenting stress is usually focused on samples from middle-class neighborhoods [48,51,52]. However, there is less empirical evidence of the consequences of parenting stress in risk neighborhoods [53,54]. Parenting stress and its consequences for children, but also for parents, cannot be the same in risk neighborhoods [47] compared to middle-class neighborhoods [51].

Overall, some studies focused on parental stress attempted to analyze its relationship with internal and external problems in children [9,28], particularly in the early stages of development when the child is more vulnerable [15]. However, less common is the use of samples from an ample age range of children across a parental socialization period (from birthhood to when the adolescent reached adult age) [55,56], in which parents have the main responsibility of raising their children in order for them to become responsible members of society [57,58]. Raising children, particularly in risk neighborhoods [59], could lead to high levels of stress in parents.

Most studies examine parenting stress from a child-centered approach; fewer studies are focused on a parent-centered approach. It could be important to analyze the relationship between parenting stress and parental adjustment due to some reasons. Firstly, parents have a greater responsibility for the physical, psychological, and emotional development of their children [19]. Second, family prevention programs are primarily aimed at parents and provide basic educational tools to increase protective behavior and reduce risk familiar factors [12,28].

This study is focused on parents of children from 1 to 18 years from Chile located in a vulnerable social environment. The present study aims to analyze the relationship between multidimensional parenting stress (i.e., parental distress, parent–child dysfunctional interaction and difficult child) and parental adjustment (i.e., depression, anxiety, and stress) in risk neighborhoods.

## 2. Materials and Methods

### 2.1. Participants and Procedure

An a priori power analysis was conducted, as in previous family studies [60,61,62]. A priori power analysis determined that 119 participants were required to detect an unfavorable medium effect size (*f* = 0.15) with a power of 0.95 (α = 0.05, 1 − β = 0.95) for linear multiple regression. Participants were 142 parents/caregivers (72 females and 70 males) of children aged from 1 to 18 years (*M* = 9.76, *SD* = 4.54) who were users of the Depending Focused Prevention Programs of the National Service for Minors in the Los Lagos Region (Chile). These parents were referred from family courts as a protection measure since their children were victims of parental neglect (23.9%), witnesses and/or victims of intra-family violence (13.4%), psychological abuse (3.5%) or physical abuse (2.1%), among other problems. The power sensibility test [63,64] for the sample size of this study (*n* = 142) was 0.95 (α = 0.05), and was able to detect a medium-small effect size (*f* = 0.12).

The demographic characteristics of the participants were as follows: Regarding most frequent marital status, most of participants were single (57.7%) in contrast to those who were married (28.2%), separated (4.2%) or widowed (2.8%). The parental figures showed a low educational level, since many caregivers only completed basic education (7%) and high school (37.3%). Regarding the socio-economic level, most of the participants (66.2%) received income from dependent and informal work activities lower than the Chilean minimum wage.

The information gathering was carried out between the months of September and December 2019. First, authorizations from the National Service for Minors the approval of the Ethics Committee of St. Sebastian University (Patagonia Headquarters, Puerto Montt, Chile) were requested to carry out the investigation with human participants. Subsequently, the invitation was extended to randomly selected caregivers to participate in the research. Once the caregivers agreed to participate in the study, they proceeded to sign the informed consent and the subsequent application of the instruments in dependencies of the Targeted Prevention Program (PPF), which was dependent on the National Service for Minors. The pen-and-paper questionnaires were filled out by participants in the presence of research assistants [65,66].

### 2.2. Measures

Parenting stress was measured with the Parenting Stress Index/Short Form (PSI/SF) [37]. It consisted of 36 items answered on a five-point scale, ranging from 1 = *strongly disagree* to 5 = *strongly agree*. The PSI/SF was designed to measure three parental stress dimensions: parental distress, parental child dysfunctional interaction and difficult child. Each dimension was measured by 12 items. The Parental Distress scale was designed to assess the amount of stress an individual was feeling as a parent due to personal factors, such as impaired parenting competence, conflict with the other parents, presence of depression, lack of social support and life restrictions due to the demands of child raising. A sample of item was “I feel trapped by my responsibilities as a parent”. The alpha value was 0.867. The Parent–Child Dysfunctional Interaction scale examined whether the parental interaction with child was seen as reinforcing to the parent or was a negative element in the parent’s life, as well as the degree to which parents perceive that the child does or does not meet his/her expectations. A sample of item was “My child rarely does things for me that make me feel good”. The alpha value was 0.897. The Difficult child subscale assessed behavioral characteristics of the child that reflected their self-regulatory abilities perceived by the parent and whether the child is difficult to manage, due either to temperamental factors or learned patterns of noncompliance and defiance. A sample of item was “My child seems to cry or fuss more often than other children “. The alpha value was 0.838. Higher scores in parental distress, parental child dysfunctional interaction and difficult child were related to higher parental stress. The Spanish version had good psychometric properties for adults [67]. The three-dimensional model, originally proposed in the Parenting Stress Index/Short Form (PSI/SF) [37], was confirmed by confirmatory factorial analysis in previous studies [49,51], including Spanish-speaking samples [68].

Parental adjustment was measured with the Depression, Anxiety and Stress Scales (DASS-21) [69], a self-report measure of negative affect symptoms in depression, anxiety and stress, its presence and intensity. It consisted of 21 items answered on a four-point Likert-type scale, ranging from 1 = *did not apply to me at all* to 4 = *applied to me very much*, *or most of the time* for weekly measures. Each dimension was measured by 7 items. The depression dimension referred to low levels of positive feelings (e.g., dysphoria, hopelessness, lack of energy, and anhedonia). A sample of depression item was “I couldn’t seem to experience any positive feeling at all”. The alpha value was 0.858. The anxiety dimension was characterized by physiological hyperarousal, a mixture of general distress such as irritability, agitation, difficulty relaxing, and impatience. A sample of anxiety item was “I was worried about situations in which I might panic and make a fool of myself”. The alpha value was 0.849. The stress dimension was related to irritability, nervous tension, difficulty relaxing, and agitation [69,70]. A sample of stress item is “I found it difficult to relax”. The alpha value was 0.833. Depression and anxiety were also related to negative feelings [71]. DASS-21 was translated and adapted in Chile [72,73] and it presented adequate psychometric properties in previous validation studies [74,75,76] and fit into a three-factor model in Spanish-speaking samples [77,78].

### 2.3. Data Analysis

A correlation analysis was performed, as well as multiple linear regression analyses. The correlation analysis was conducted between parenting stress (i.e., parental distress, parent–child dysfunctional interaction and difficult child) and parental adjustment (i.e., depression, anxiety, and stress). A lineal regression was applied in which the dependent variables were those related to parental adjustment (i.e., depression, anxiety, and stress) and the predictors were the three dimensions of parenting stress (i.e., parental distress, parent–child dysfunctional interaction and difficult child).

## 3. Results

Results from correlations analyses between parenting stress and parental adjustment are presented in Table 1. Significant associations were found among the three parental adjustment dimensions, as well as among some dimensions of parenting stress and parental adjustment. When examining correlations between parenting stress dimensions (i.e., parental distress, parent–child dysfunctional interaction and difficult child) and parental adjustment dimensions (i.e., depression, anxiety, and stress), only one parenting stress dimension (i.e., parental distress) was positively associated with all parental adjustment dimensions. By contrast, the other parenting stress dimensions were not associated with parental adjustment. Within parenting stress dimensions, only a high positive relation between parent–child dysfunctional interaction and difficult child was found. Within parental adjustment dimensions, a high positive correlation between depression, anxiety, and stress was also identified.

The results for the predictions of parental adjustment (i.e., depression, anxiety, and stress), depending on parenting stress (i.e., parental distress, parent–child dysfunctional interaction and difficult child) analyzed through a linear multiple regression analyses, are presented in Table 2. A multiple linear regression model for each dimension of parental adjustment was performed. Interestingly, a common pattern was found for the three predictions models. For the prediction of parental depression, parental distress was found as a significant predictor, but not parent–child dysfunctional interaction and difficult child. Greater parental distress positively predicts depression in parents. In the same way, for the prediction of parental anxiety, the statistically significant predictor was also parental distress. The trend for parental distress was positive; greater scores predicted parental anxiety. Finally, the same is true for the prediction of parental anxiety. Only parental distress was a significant predictor, whereas parent–child dysfunctional interaction and difficult child did not reach the statistically significant level. Overall, parental anxiety was a significant predictor of three components of parental adjustment. However, a higher explained variance was found in parental depression, in comparison to parental anxiety and stress, in which explained variance was similar.

## 4. Discussion

This study analyzed the relationship between parenting stress (i.e., parental distress, parental child dysfunctional interaction and difficult child) and parental adjustment (i.e., depression, anxiety, and stress) in a sample of parents of children aged from 1 to 18 years and located in vulnerable social environments in Chile. These children were in situations where their rights were violated, in adoption processes and in conflict with the law; present findings were provided from a clinical sample. Overall, the present results showed that only one parenting stress dimension (i.e., parental distress) was positively associated with poor parental adjustment in terms of greater depression, anxiety, and stress. Interestingly, not all components of parental stress are significant predictors of parental adjustment. The present findings revealed that the parental adjustment of children in vulnerable social contexts could only be predicted by parental distress, but not by parent–child dysfunctional interaction and difficult child.

The present findings agree with some previous studies on the negative relationship between parenting stress and health/well-being, most of them from middle-class community samples. Parenting stress has a negative impact on child raising, deteriorating the quality of the bond and the deployment of effective skills and tools for the care and protection of children. Parenting stress seems to negatively influence child development [79], expressed in higher levels of behavioral maladjustment problems and child negativity [10,80,81,82,83]. A greater perception of stress in parents is associated with more childhood problems, deteriorating parenting and the quality of the bond between father and son [84,85,86]. A possible explanation could be that a child’s sense of security is affected by parental stress, especially when parents have difficulties regulating their own emotions or maintaining timely, safe family routines that are suitable for the child [87].

However, compared to those studies on parenting stress and its relationship with child development [9,28,44,47], less is known about the relationship between parental stress and parental health. The findings from the present study agreed with some previous studies on the harmful impact of parenting stress [88,89]. For example, those parents who presented high levels of parental stress when raising their children showed a higher incidence of depression and anxiety [11,90] and lower levels of general well-being [91]. Parenting stress is often associated with a greater deterioration in parental mental health [92], evidencing a greater risk of postpartum depression [93,94]. Parenting stress was related to depression, anxiety and stress in mothers of 10-year-old children from a community sample [46]. Other studies reported that parenting stress negatively influences parental competence, such as sensitivity to the needs of the child, the time and quality of parenting, dyadic pleasure and cooperation between parents [95].

An important focus is the analysis of the consequences of parenting stress based on a single variable (i.e., global dimension) or with its specific components (i.e., multidimensional approach). The same is true for other family and personal variables such as parenting dimensions [5,96] and styles [97], self-esteem [98,99] and self-concepts [100,101], family climate [102] and psychosocial maturity [103]. Some studies identified relations between adjustment and parenting stress from a unidimensional perspective [46,47,48]. For example, global parenting stress was positively related to parental maladjustment (caused by depression, anxiety and stress) [46] and greater child maladjustment (caused by traumatic life events and depression) [47].

Nevertheless, when parenting stress is examined using a multidimensional approach, not all of its different components (i.e., parental distress, parent–child dysfunctional interaction and difficult child) are equally related to the different adjustment criteria examined [49,50]. For example, findings from a study with African American mothers showed that the three parenting stress dimensions examined (i.e., parental distress, parent–child dysfunctional interaction and difficult child) were not equally related to different parental adjustment criteria [49]. Specifically, parental distress was related to self-reported psychological symptoms, parent–child dysfunctional interaction was associated with parent psychological symptoms, and difficult child was most highly associated with a measure of child oppositionality. Similar findings were obtained in a study based on path analysis. Interestingly, for both fathers and mothers, parental distress was positively related to potential parental abuse, whereas parent–child dysfunctional interaction and difficult child did not show a relationship [50].

Additionally, parenting stress may not always be the same for families from vulnerable environments [47,53,54] compared to those from different experiences, i.e., children raised in middle class neighborhoods [48,51,52]. The results obtained in the present study are partially related to the theories on family and parental stress (see [38,104]). The cumulative impact of the different psychosocial stressors for families, especially those related to child rearing (e.g., daily discomfort) could be related to an appearance of higher levels of parental stress, particularly greater in risk neighborhoods in which families, schools, and jobs offer less opportunities for healthy development. In a general risk context for individuals and families, in which daily life is related to a greater general stress, the influences of the different components of parenting stress on adjustment (for children, but also for parents) could not have the same impact as in different contexts [105].

This study has strengths and limitations. The present study examined the relationship between parenting stress and parent psychological health, offering new and crucial evidence of the harmful impact of parenting stress for parents and not simply for children, as was more common in previous studies [84,85]. Furthermore, the present study extends the evidence of parenting stress to vulnerable families from risk neighborhoods. Compared to the classical unidimensional (i.e., global) approach to examining parenting stress, the present study highlights that which component of parenting stress was related to each adjustment criteria should be empirically examined. Interestingly, only parental distress but not parent–child dysfunctional interaction and difficult child are significant predictors of poor parental adjustment. Additionally, the sample size of the present study was determined by a statistical power analysis. However, some limitations should be considered. The present study, with correlation and regression analyses, does not determine a relationship of causality between parenting stress and parental adjustment variables. The present study was conducted with a clinical sample of parents in a risk neighborhood from Chile, but it was not possible to conduct more studies in other settings to extend the findings on parenting stress to other contexts, particularly the study of which components of parenting stress were related to each adjustment criteria.

Raising children in risk neighborhoods involves many psychosocial stressors that did not appear in community samples, such as poverty, marginalization, unemployment, early experiences of child abuse, domestic violence [106,107], and the problematic consumption of alcohol and drugs. Some studies with vulnerable communities showed that there was a greater stress in risk neighborhoods such as families with children with disabilities [108,109], language disorders, autism or behavioral problems [109]. The impact of these stressors on parental adjustment could be different in community samples than in risk neighborhoods. Parenting distress could affect parental and child adjustment and high levels of parenting distress in risk neighborhoods could be related to poor parental adjustment more than in community samples.

The relation between stressors in risk neighborhoods and parenting stress, and its relation with parental adjustment, is a relevant area of study that could have repercussions on the future development of a child [13], and developing interventions that focus on child protection and adjustment, as well as supporting mental health disorders in parents, should be considered. It is important to keep in mind that the levels of parenting stress could vary depending on the parental perception of the situations and their coping strategies and highlighted the importance of the first interventions, education and support for parents and caregivers of children with disabilities and developing disorders [108]. Similarly, the detection of the deficits in the competence of parents, and the parental implications of this, would allow timely, systematic, and coherent intervention mechanisms to be implemented, considering the needs and requirements of the family, particularly when they are located in vulnerable contexts, and it could be utilized from a public health, legal and psychosocial perspective.

## 5. Conclusions

This study showed that parenting stress could be related to parental adjustment and provides empirical evidence of the different components of parenting stress and which of its components are more frequently related to parental adjustment in vulnerable social contexts from Chile (i.e., parental distress, but not parent–child dysfunctional interaction and difficult child). The present findings were derived from a clinical sample of parents who raised children in a risk neighborhood. It should be taken into account that, in vulnerable contexts, there is a daily exposure to psychosocial stressors, so the relations between the different components of parenting stress and adjustment could not have the same impact as in different contexts. In the present study, there is a multidimensional approach of parenting stress (i.e., parental distress, parent–child dysfunctional interaction and difficult child). A multidimensional approach reveals which component of parenting stress is related to each adjustment criteria, which is impossible to determine using a unidimensional approach of parenting stress. Parenting stress has a negative impact on parent and child adjustment; therefore, it is important to detect parenting stress and its dimensions and the different impacts on child and parent adjustment. It is important to keep this in mind when developing interventions with parents, as parental stress may affect the psychological health of parents and this could interfere with raising children.

## Figures and Tables

**Table 1:** Correlations between parenting stress and parental adjustment.

		Parenting Stress			Parental Adjustment		
		Parental Distress	Parent–Child Dysfunctional Interaction	Difficult Child	Depression	Anxiety	Stress
Parenting stress	Parental distress	1					
	Parent–child dysfunctional interaction	0.069	1				
	Difficult child	−0.043	0.732 **	1			
Parental adjustment	Depression	0.821 **	0.13	0.009	1		
	Anxiety	0.637 **	0.059	−0.013	0.757 **	1	
	Stress	0.565 **	0.057	−0.081	0.772 **	0.739 **	1

** *p* < 0.01.

**Table 2:** Multiple linear regression coefficients between parenting stress and parental adjustment.

Dependent Variable	Predictors	*B*	SE B	β	*t*	Lower	Upper
Parental depression *R^2^adj* = 0.673 *F*(3, 138) = 97.83 ***	Parental Distress	0.68	0.04	0.81	16.71 ***	0.595	0.755
	Parent–child Dysfunctional Interaction	0.07	0.06	0.09	1.26	−0.040	0.182
	Difficult child	−0.02	0.06	−0.02	−0.30	−0.134	0.099
Parental anxiety *R^2^adj* = 0.393 *F*(3, 138) = 31.45 ***	Parental Distress	0.53	0.06	0.64	9.59 ***	0.417	0.633
	Parent–child Dysfunctional Interaction	0.01	0.08	0.01	0.09	−0.144	0.157
	Difficult child	0.01	0.08	0.01	0.09	−0.151	0.165
Parental stress *R^2^adj* = 0.316 *F*(3, 138) = 22.70 ***	Parental Distress	0.49	0.06	0.55	7.79 ***	0.364	0.611
	Parent–child Dysfunctional Interaction	0.11	0.09	0.13	1.27	−0.061	0.283
	Difficult child	−0.14	0.09	−0.15	−1.49	−0.316	0.044

*** *p* < 0.001.

## Data Availability

All data are available in this manuscript.
